# MMDD: A Multimodal Multitask Dynamic Disentanglement Framework for Robust Major Depressive Disorder Diagnosis Across Neuroimaging Sites

**DOI:** 10.3390/diagnostics15233089

**Published:** 2025-12-04

**Authors:** Qiongpu Chen, Peishan Dai, Kaineng Huang, Ting Hu, Shenghui Liao

**Affiliations:** School of Computer Science and Engineering, Central South University, Changsha 410083, China; 234711045@csu.edu.cn (Q.C.); 234711053@csu.edu.cn (K.H.); 234712249@csu.edu.cn (T.H.); lsh@csu.edu.cn (S.L.)

**Keywords:** multimodal learning, magnetic resonance imaging, multitask learning, multisite collaboration, major depressive disorder, deep learning

## Abstract

**Background/Objectives:** Major Depressive Disorder (MDD) is a severe psychiatric disorder, and effective, efficient automated diagnostic approaches are urgently needed. Traditional methods for assessing MDD face three key challenges: reliance on predefined features, inadequate handling of multi-site data heterogeneity, and suboptimal feature fusion. To address these issues, this study proposes the Multimodal Multitask Dynamic Disentanglement (MMDD) Framework. **Methods:** The MMDD Framework has three core innovations. First, it adopts a dual-pathway feature extraction architecture combining a 3D ResNet for modeling gray matter volume (GMV) data and an LSTM–Transformer for processing time series data. Second, it includes a Bidirectional Cross-Attention Fusion (BCAF) mechanism for dynamic feature alignment and complementary integration. Third, it uses a Gradient Reversal Layer-based Multitask Learning (GRL-MTL) strategy for enhancing the model’s domain generalization capability. **Results:** MMDD achieved 77.76% classification accuracy on the REST-meta-MDD dataset. Ablation studies confirmed that both the BCAF mechanism and GRL-MTL strategy played critical roles: the former optimized multimodal fusion, while the latter effectively mitigated site-related heterogeneity. Through interpretability analysis, we identified distinct neurobiological patterns: time series were primarily localized to subcortical hubs and the cerebellum, whereas GMV mainly involved higher-order cognitive and emotion-regulation cortices. Notably, the middle cingulate gyrus showed consistent abnormalities across both imaging modalities. **Conclusions:** This study makes two major contributions. First, we develop a robust and generalizable computational framework for objective MDD diagnosis by effectively leveraging multimodal data. Second, we provide data-driven insights into MDD’s distinct neuropathological processes, thereby advancing our understanding of the disorder.

## 1. Introduction

Major Depressive Disorder (MDD) is a severe mental illness characterized by persistent low mood, loss of interest, and recurrent suicidal thoughts, significantly impairing patient quality of life [1]. According to the latest epidemiological data from the World Health Organization (WHO), approximately 280 million people worldwide suffer from depression, making it one of the leading causes of disability worldwide [2]. However, the diagnosis of MDD primarily relies on clinicians’ subjective evaluation of patients’ symptoms. These diagnostic approaches depend largely on patients’ self-reports and physicians’ clinical experience and lack objective biomarkers. As a result, diagnostic consistency among different doctors is low. Moreover, MDD’s symptom spectrum overlaps significantly with other psychiatric disorders. This further increases the difficulty of early differential diagnosis [3]. Although a growing body of research has explored potential biomarkers for MDD [4], the field has not yet converged on a standardized set of criteria for clinical application. Thus, developing an objective and quantifiable biomarker system is critical, and has therefore become an urgent challenge in psychiatry.

Structural magnetic resonance imaging (sMRI) and functional magnetic resonance imaging (fMRI) are core components of neuroimaging. They provide complementary objective evidence for the pathological mechanisms of MDD. Specifically, sMRI focuses on brain morphology [5], while fMRI focuses on dynamic neural activity [6,7]. sMRI, through high-resolution brain scanning, enables the quantification of structural metrics such as gray matter volume (GMV), white matter volume, and cerebrospinal fluid. Research has consistently demonstrated that MDD patients often exhibit structural abnormalities in brain regions including the prefrontal cortex and occipital lobe [8,9]. These alterations may be associated with neuroplasticity impairment resulting from chronic mood disorders. fMRI captures temporal variations in blood-oxygen-level-dependent (BOLD) signals, reflecting functional connectivity (FC) patterns and neural network dynamics during both resting-state and task-based conditions. For instance, functional abnormalities in the default mode network (DMN) have been conclusively linked to MDD’s negative cognitive biases and emotional dysregulation [10,11]. These analyses show that sMRI and fMRI offer complementary insights into MDD, together providing an objective perspective on its neurobiological basis. Nevertheless, transforming these information-rich imaging datasets into clinically applicable diagnostic tools still requires overcoming a series of technical bottlenecks.

Machine learning has become a pivotal tool in biomedical research. Its impactful applications range from the accurate prediction of diabetes [12] to the management of hematological disorders [13], demonstrating its broad utility. In the specific domain of psychiatric neuroimaging, however, translating these insights into clinically applicable diagnostic tools for MDD faces three major challenges: data harmonization, model construction, and multimodal fusion. First, data harmonization remains a significant hurdle. While multi-center studies expand sample sizes, the resulting data often exhibit significant site heterogeneity due to variations in MRI scanners, sequence parameters, and acquisition protocols. This heterogeneity introduces systematic biases that can confound disease-related signals. Existing harmonization methods like ComBat [14] typically assume site effects are independent of biological signals, representing a static correction approach. Although recent frameworks such as $AD^{2}A$ [15], USMDA [16], and FSM-MSDA [17] have advanced site adaptation via adversarial learning and domain adaptation, they are primarily validated on a limited number of sites (e.g., 2–3 centers) and face scalability and stability challenges when applied to large-scale multi-center settings involving 25 sites. Second, effective model construction for multimodal MRI data demands a paradigm shift from generic architectures to designs that explicitly encode modality-specific features. MRI data exhibits high-dimensional characteristics and inherent noise (e.g., head motion artifacts, field inhomogeneity), posing challenges such as the curse of dimensionality and feature extraction bottlenecks for conventional machine learning methods. Traditional analytical approaches rely on predefined imaging features (e.g., functional connectivity [11,18]) and shallow machine learning models (e.g., support vector machines, linear regression [10,19]), which are ill-suited to capture the complex nonlinear pathological patterns of MDD. Current research tends to directly model raw signals through end-to-end deep learning frameworks (e.g., convolutional neural networks (CNNs) [20,21], Swin Transformers [22,23]). While these approaches avoid the limitations of handcrafted feature design, they do not address the critical need for modeling modality-specific characteristics. Third, achieving deep multimodal fusion is nontrivial. Most existing methods adopt simple concatenation or traditional cross-attention fusion strategies. Moreover, they fail to fully explore the deep correlations between structural and functional modalities. Traditional cross-attention fusion methods [24] typically use a unidirectional mechanism: one modality serves as the Query, while the other acts as the Key and Value, forming a unidirectional information flow. The main flaws of this design lie in asymmetric information flow and insufficient modal interaction.

To overcome these challenges, we propose the Multimodal Multitask Dynamic Disentanglement Framework (MMDD). The key contributions of this work are outlined below:

- To tackle the critical issue of site heterogeneity in large-scale multi-center MDD studies, we propose a Gradient Reversal Layer-based Multitask Learning (GRL-MTL) strategy. Instead of applying a static correction, our method reframes site harmonization as an auxiliary task. By leveraging a Gradient Reversal Layer, it dynamically learns site-invariant representations that are robust to scanner and protocol variations. This approach demonstrates superior scalability and stability in experiments involving a large number of sites.
- We designed a dual-pathway feature extraction network to accurately capture modality-specific characteristics from sMRI and fMRI data. Specifically, a 3D ResNet is employed to extract rich spatial structural features from sMRI, while an LSTM–Transformer hybrid encoder is designed to model the complex temporal dynamics of fMRI. This specialized design ensures the optimal extraction and representation of each modality’s unique information before fusion, forming a more informed basis for multimodal integration.
- For the core challenge of multimodal integration, we introduce a Bidirectional Cross-Attention Fusion (BCAF) mechanism. Unlike traditional unidirectional fusion methods, BCAF establishes bidirectional interaction paths, allowing sMRI and fMRI to mutually and simultaneously guide each other’s representation learning. This process dynamically integrates complementary information through attention-based weighting, thus forming a more holistic and discriminative feature representation.
- On the REST-meta-MDD dataset (comprising 1300 MDD patients and 1128 healthy controls across 25 sites), our model achieved an overall classification accuracy of 77.76%. In a more challenging leave-one-site-out cross-validation setting (using all available sites as independent test sets), our method consistently outperformed baseline approaches across all test sites, demonstrating strong generalizability across unseen sites.
- Interpretability analysis, based on SHAP values and two-sample *t*-tests, revealed distinct neurobiological patterns: temporal features from fMRI were predominantly localized to subcortical hubs and the cerebellum, while gray matter volume (GMV) features mainly involved higher-order cognitive and emotion-regulation cortical regions. Notably, the middle cingulate gyrus consistently exhibited significant abnormalities in both imaging modalities.

## 2. Related Work

Based on the multi-center REST-meta-MDD dataset, computer-aided diagnosis of MDD using resting-state fMRI (rs-fMRI) has become an important research direction. Existing work primarily revolves around the core challenge of effectively modeling brain FC using Graph Neural Networks (GNNs). It has evolved from static FC extraction to dynamic FC generation, and from a general architecture to a specifically optimized one. Several recent studies (2023–2025) on MRI-based MDD classification were selected for analysis in the review, as summarized in Table 1. Early research, such as DGCNN [25], initially validated the effectiveness of GNN in processing static FC. DGCNN achieved performance superior to traditional machine learning methods (with an accuracy of 72.1%) on a sample of 1601 subjects, laying the foundation for subsequent studies. However, research by Gallo et al. [26] demonstrated that simply applying standard Graph Convolutional Network (GCN) or support vector machine (SVM) models leads to a significant decline in generalization performance (62% accuracy) on large multi-center datasets (2338 subjects) due to site heterogeneity. To address this challenge, the FGDN [27] framework integrates linear and nonlinear functional connectivity information through its Dual Graph Attention Network (DGAT) module to extract more robust features. More importantly, it reconstructs the disrupted graph structural connections across different sites via the Federated Graph Convolutional Network (FedGCN) module. However, its classification accuracy of 61.8% on 841 subjects from three sites of the REST-meta-MDD dataset indicates that multi-site depression diagnosis still faces significant challenges. This highlights the necessity for model innovation.

Subsequent researchers have deepened this work along multiple dimensions, although validation is often not performed on the complete dataset. Firstly, in terms of feature learning, GAE-FCNN [18] learns low-dimensional topological embeddings of brain networks in an unsupervised manner, aiming to more fully capture complex relationships between nodes. The N2V-GAT [28] framework innovatively combines Node2Vec graph embedding with graph attention mechanisms to differentially evaluate the contributions of different brain regions, achieving high classification performance (78.73% accuracy). Secondly, on the data modeling level, research expanded from static to dynamic networks. For instance, the DSFGNN [29] framework attempts to capture temporal dynamics of brain activity by simultaneously modeling static and dynamic FC and fusing them using an attention mechanism. Meanwhile, BrainFC-CGAN [30] adopts a generative approach, specifically designing layers to preserve the symmetry and topological properties of FC. Thirdly, regarding data quality, DDN-Net [31] directly addresses noise in fMRI data by introducing deep residual shrinkage denoising and subgraph normalization, enhancing model robustness. Although the aforementioned methods have advanced MDD classification, they typically rely on predefined brain atlases to extract FC as input, which carries potential information loss. This limitation is also evident in broader neuroimaging research.

The current trend is to bypass handcrafted feature extraction and perform end-to-end learning directly on raw signals. For example, Muksimova et al. [20] proposed a CNN architecture integrated with an attention mechanism for end-to-end learning from original MRI slices, enabling automatic multi-scale feature capture and fusion. Xin et al. [22] employed a CNN + Swin Transformer dual-stream network to process static 3D sMRI, focusing on capturing spatial patterns of gray/white matter atrophy. SwiFT [23] adapted the Swin Transformer for dynamic 4D fMRI analysis by introducing a temporal modeling mechanism, enabling dynamic characterization of FC networks. These methods focus on single modality. Despite significant progress in deep learning-based MRI analysis, comprehensively parsing the complex human brain often requires integrating multimodal data.

Multimodal medical imaging research has made remarkable progress [32,33,34], focusing on integrating data from different modalities to enhance model perception, understanding, and reasoning. Early studies like camAD [24] used CNN-based multi-scale feature extraction and cross-attention fusion, dynamically weighting different modalities to significantly improve Alzheimer’s disease classification accuracy. Similarly, AGGN [35] further developed a dual-domain (channel + spatial) attention mechanism combined with multi-scale feature extraction for precise glioma grading. In terms of architectural innovation, OmniVec2 [36] built a universal Transformer framework supporting 12 modalities, using cross-attention in a dual-stream architecture for intermodal feature fusion and enabling cross-modal knowledge transfer via shared transformer parameters. To improve computational efficiency, MDDMamba [37] introduced the CrossMamba module, replacing the traditional Transformer’s quadratic complexity with a linear alternative for efficient multimodal fusion. For modeling high-order relationships, CIA-HGCN [38] applied hypergraph convolutional networks to mental disorder classification, capturing complex biomarker associations by constructing brain region-gene hypergraph networks. These methods collectively drive the paradigm shift in multimodal analysis from simple feature concatenation to deep, intelligent fusion, focusing on uncovering latent biological correlations through adaptive mechanisms.

Site heterogeneity in multi-center data poses a significant challenge, as existing static harmonization methods struggle to dynamically disentangle site-specific variances from disease biomarkers. Multi-site neuroimaging has become a key paradigm, enhancing statistical power and generalizability by aggregating data from different scanners and populations. Early methods focused on data-level harmonization; for instance, ComBat [14], which uses an empirical Bayes framework for static correction of site effects, became a widely used baseline. Subsequently, SiMix [39] introduced data augmentation via cross-site image mixing and test-time multi-view boosting to improve generalization to unseen sites. With the rise in deep learning, Svanera et al. [40] used a progressive level-of-detail network, learning site-invariant anatomical priors at lower levels while adaptively handling site-specific intensity distributions at higher levels, achieving implicit heterogeneity disentanglement. The domain adaptation paradigm spurred advances in feature-space alignment techniques: Guan et al. [15] used an adversarial dual-branch structure to separate shared and site-specific features; USMDA [16] enabled unsupervised multi-source domain adaptation; FSM-MSDA [17] established semantic feature matching mechanisms. Recent research also addresses privacy preservation and heterogeneous collaboration: SFPGCL [41] innovatively combined federated learning with contrastive learning, using a shared branch to capture global invariant features and a personalized branch to retain site-specific characteristics, mitigating client heterogeneity via contrastive learning; SAN-Net [42] employed self-adaptive normalization and Gradient Reversal Layers to dynamically suppress site-related variations at the feature level.

In summary, research on automated MDD diagnosis and MRI analysis clearly illustrates a technological development path from methodological innovation to addressing real-world challenges. Initially, various neural network models significantly enhanced the characterization of brain functional connectivity networks by incorporating attention mechanisms, dynamic fusion, and embedding learning. However, the inherent site heterogeneity and scanner effects in multi-center data have highlighted the necessity of moving beyond mere model performance optimization. This has driven the development of technologies such as domain adaptation, feature disentanglement, and federated learning, aimed at enhancing model generalizability and robustness. Looking ahead, research in this field will increasingly focus on integrating the depth of end-to-end learning with the breadth of multimodal fusion, while leveraging explainable artificial intelligence technologies to ensure model transparency and trustworthiness. Ultimately, through the synergistic optimization of algorithmic architecture, data harmonization, and multimodal information integration, we can expect to develop objective diagnostic tools that are truly applicable in clinical practice and demonstrate high generalizability.

**Table 1:** Recent research on MRI-based MDD classification on the REST-meta-MDD dataset.

Study	Methodology	Sample	Data Characteristics	Site Harmonization	Accuracy
Zhu et al. (2023) [25]	Deep Graph CNN	HC, *n* = 771 MDD, *n* = 830	fMRI	No (Random split)	72.10%
Gallo et al. (2023) [26]	SVM and GCN	HC, *n* = 1083 MDD, *n* = 1255	fMRI	No (Random split)	62.00%
Zheng et al. (2023) [43]	a brain function encoder and a brain structure encoder to extract features, a function and structure co-attention fusion module	HC, *n* = 1179 MDD, *n* = 1008	fMRI and sMRI	No (Random split)	75.20%
Noman et al. (2024) [18]	graph autoencoder (GAE) architecture, built upon GCN	HC, *n* = 227 MDD, *n* = 250	fMRI	No (Random split)	65.07%
Tan et al. (2024) [30]	Generative Adversarial Network (GAN)	HC, *n* = 684 MDD, *n* = 747	fMRI	No (Random split)	76.28%
Zhao et al. (2024) [29]	GNN	HC, *n* = 779 MDD, *n* = 832	fMRI	No (Random split)	67.12%
Zhang et al. (2024) [31]	Deep Residual Shrinkage Denoising Network	unknown	fMRI	No (Random split)	72.43%
Xi et al. (2025) [44]	a Random Forest and an ensemble classifier	HC, *n* = 742 MDD, *n* = 821	fMRI	No (Random split)	75.30%
Su et al. (2025) [28]	Graph Attention Network (GAT)	HC, *n* = 765 MDD, *n* = 821	fMRI	No (Random split)	78.73%
Liu et al. (2025) [27]	a Federated Graph Convolutional Network framework with Dual Graph Attention Network	HC, *n* = 384 MDD, *n* = 457	fMRI	Yes	61.80%

## 3. Materials and Methods

**Figure 1:**
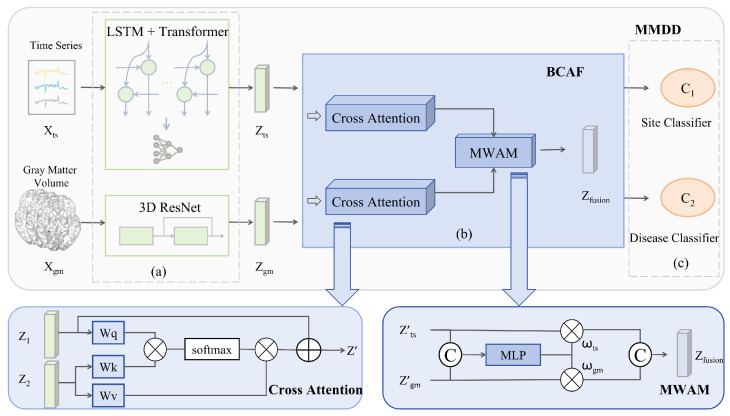
Architecture of the proposed MMDD framework for MDD classification. The framework comprises three main stages: (**a**) a dual-pathway feature extraction module; (**b**) a Bidirectional Cross-Attention Fusion (BCAF) module; and (**c**) a joint classification module based on a Gradient Reversal Layer Multitask Learning (GRL-MTL) strategy.

We propose a coordinated multi-site multimodal multitask learning framework that synergistically integrates time series data and gray matter volume (GMV) data to achieve accurate classification of brain disorders (the model architecture is shown in Figure 1). MMDD consists of two parallel modality-specific pathways to process individual modality inputs, followed by a cross-attention mechanism for adaptive intermodal information sharing. After obtaining the fused representation, we implement a dual-task learning scheme consisting of the following: (1) primary task: binary classification between MDD patients and healthy controls (HCs); (2) auxiliary task: site classifier. Concurrently, the model is trained using a Gradient Reversal Layer (GRL) in an adversarial learning manner, to eliminate site-specific characteristics from the features, thereby learning site-invariant feature representations.

### 3.1. Dual-Pathway Feature Extraction

In the temporal feature extraction branch, we combine the model structures of bidirectional long short-term memory (BiLSTM) [45] and Transformer encoder [46]. The input time series data is represented as $X_{ts}\in R^{B\times T\times d}$, where B denotes batch size, T represents time steps, and d indicates feature dimensionality. To handle variable-length sequences, we apply zero-padding to standardize the sequence lengths and generate a corresponding binary mask $M\in {\left\{0,1\right\}}^{B\times T\times d}$ to identify valid time steps. The branch processes the sequences through a BiLSTM layer as follows: the forward LSTM processes the sequence from start to end as shown in Equation (1), while the backward LSTM processes it in reverse order as shown in Equation (2). The complete BiLSTM output $H_{lstm}$ is obtained by concatenating both directional outputs according to Equation (3).

(1) $$\vec{h_{t}}=\mathrm{LSTM}\left(x_{ts},\vec{h_{t-1}}\right)$$

(2) $$\overset{\leftarrow }{h_{t}}=\mathrm{LSTM}\left(x_{ts},\overset{\leftarrow }{h_{t-1}}\right)$$

(3) $$H_{lstm}=\left[\vec{h}\|\overset{\leftarrow }{h}\right]\in R^{B\times T\times 2d}$$

where, $\vec{h_{t}}$ and $\overset{\leftarrow }{h_{t}}$ represent the forward and backward hidden states, respectively, and “‖” indicates feature concatenation.

The Transformer encoder layer disregards the zero-padded positions, enabling global modeling of temporal dependencies. We perform a weighted average of the Transformer encoder outputs based on the input mask, ultimately obtaining high-level temporal sequence feature representations $H_{\mathrm{trans}}$ as defined in Equation (4). This representation is subsequently transformed into the final temporal feature vector $z_{ts}$ through a linear projection layer, formulated in Equation (5).

(4) $$H_{\mathrm{trans}}=\mathrm{TransformerEncoder}\left(H_{\mathrm{lstm}}\right)$$

(5) $$z_{ts}=W_{0}\;\cdot \;\left(\frac{1}{T}\sum_{t=1}^{T}H_{\mathrm{trans}}\right)+b_{0}$$

where, $W_{0}$ denotes the learnable weight parameters and b represents the bias terms. In the GMV feature extraction branch, we employ a 3D ResNet [47] architecture for hierarchical spatial feature learning. GMV is represented as $X_{gm}\in R^{B\times 1\times D\times H\times W}$, where the spatial dimensions D×H×W correspond to the depth, height and width of the 3D brain volume, respectively. This branch processes the volumetric input through four cascaded residual blocks $\left({⨁}_{l=1}\right)$, with each stage containing residual units. The final GMV representation is computed as shown in Equation (6):

(6) $$z_{gm}=\mathrm{Softmax}\left(W_{2}\;\cdot \;\left({∥{\mathrm{ResBlock}}_{l}^{⊗n_{l}}\left(x_{gm}\right)∥}_{l=1}^{4}\right)+b_{2}\right)$$

where Softmax(·) is the softmax activation function; ${\|}_{l=1}^{4}$ denotes the concatenation of features from the four residual block groups; and the superscript $⊗n_{l}$ denotes that the l-th residual block is stacked sequentially $n_{l}$ times.

### 3.2. Bidirectional Cross-Attention Fusion

We propose the Bidirectional Cross-Attention Fusion (BCAF) for integrating deep temporal features with structural features. The first stage employs a cross-attention mechanism, achieving dynamic alignment between functional signals and anatomical foundations through symmetric attention computation. Taking the time series branch as an example, the cross-attention from time series to GMV is calculated as shown in Equation (7), and the updated temporal feature $z_{ts}^{\prime }$ is obtained via Equation (8). The structural branch follows the same computation symmetrically to yield $z_{gm}^{\prime }$.

(7) $${\mathrm{Attention}}_{ts\to gm}=\mathrm{Softmax}\left(\frac{\left(z_{ts}W_{Q}^{1}\right){\left(z_{gm}W_{K}^{1}\right)}^{T}}{\sqrt{d_{in}}}\right)$$

(8) $$z_{ts}^{\prime }=z_{ts}+{\mathrm{Attention}}_{ts\to gm}\;\cdot \;\left(z_{gm}W_{V}^{1}\right)$$

where, $z_{ts}W_{Q}^{1}$ serves as the Query; $z_{gm}W_{K}^{1}$ and $z_{gm}W_{V}^{1}$ serve as the Key and Value; the scaling factor $1/\sqrt{d_{in}}$ is introduced to stabilize gradient propagation. Symmetrically, the $z_{gm}^{\prime }$ is computed in a mirrored manner.

The second stage is implemented through a Modality Weighting Allocation Module (MWAM). The calculation formulas are shown in Equation (9) and (10).

(9) $$\omega =\mathrm{Softmax}\left(W_{4}\;\cdot \;\mathrm{ReLU}\left(W_{3}\left(z_{ts}^{\prime }\|z_{gm}^{\prime }\right)\right)\right)$$

(10) $$z_{\mathrm{fusion}}=\omega_{1}z_{ts}^{\prime }+\omega_{2}z_{gm}^{\prime }$$

where the symbol ‖ denotes the vector concatenation operation, and ReLU is the rectified linear unit activation function, defined as ReLU(x) = max(0,x). Algorithm 1 summarizes the complete flow of the proposed MMDD model.
**Algorithm 1** Proposed MMDD Framework for MDD Classification  1: **Input:** Time series data $X_{\mathrm{ts}}\in R^{B\times T\times d}$ (batch size *B*, time steps *T*, feature dim *d*), GMV data $X_{\mathrm{gm}}\in R^{B\times 1\times D\times W\times H}$(batch *B*, channel 1, depth *D*, width *W*, height *H*), ground truth labels $y_{D}\in {\left\{0,1\right\}}^{B}$, site information $y_{S}\in {\left\{0,\ldots ,24\right\}}^{B}$
  2: **Output:** Prediction probability ${\hat{y}}_{D}$ for MDD
  3: 
  4: **Step 1: Time Series Feature Extraction**  5: Generate padding mask $M\in {\left\{0,1\right\}}^{B\times T}$ for variable-length sequences  6: $H_{\mathrm{lstm}}\leftarrow \mathrm{BiLSTM}\left(X_{\mathrm{ts}}\right)$$▷H_{\mathrm{lstm}}\in R^{B\times T\times 2d}$  7: **for** l=1 **to** $L_{\mathrm{trans}}$ **do**▷$L_{\mathrm{trans}}$ = 4  8:     $H_{l}\leftarrow \mathrm{TransformerEncoderLayer}\left(H_{l-1},M\right)$▷$H_{0}=H_{\mathrm{lstm}}$  9: **end for**10: $Z_{\mathrm{ts}}\leftarrow W_{1}\;\cdot \;\mathrm{GlobalAvgPool}\left(H_{L}\right)+b_{1}$▷ Final time series features11:   
12: **Step 2: GMV Feature Extraction**13: $F_{0}\leftarrow X_{\mathrm{gm}}$14: **for** l=1 to $L_{\mathrm{res}}$ **do**▷ Process each block, $L_{\mathrm{res}}$ = 415:     $F_{l}\leftarrow \mathrm{ResNet}3\mathrm{DBlock}\left(F_{l\text{-}1}\right)$16: **end for**17: $Z_{\mathrm{gm}}\leftarrow W_{2}\;\cdot \;\mathrm{AdaptiveAvgPool}\left(F_{L}\right)+b_{2}$▷ Final GMV features18:   
19: **Step 3: Bidirectional Cross-attention Fusion**20: $Z_{\mathrm{ts}}^{\prime }\leftarrow Z_{\mathrm{ts}}+\mathrm{CrossAttention}\left(Q=Z_{\mathrm{ts}},K=V=Z_{\mathrm{gm}}\right)$21: $Z_{\mathrm{gm}}^{\prime }\leftarrow Z_{\mathrm{gm}}+\mathrm{CrossAttention}\left(Q=Z_{\mathrm{gm}},K=V=Z_{\mathrm{ts}}\right)$22: $\omega \leftarrow \mathrm{Softmax}(W_{c}\;\cdot \;\mathrm{ReLU}\left(W_{a}\left[Z_{\mathrm{ts}}^{\prime }∥Z_{\mathrm{gm}}^{\prime }\right]\right))$▷$\omega \in R^{B\times 2}$, [·∥·] is concatenation23: $Z_{\mathrm{fusion}}\leftarrow \omega \left[:,0\right]⊙Z_{\mathrm{ts}}^{\prime }+\omega \left[:,1\right]⊙Z_{\mathrm{gm}}^{\prime }$▷⊙ is element-wise multiplication24:   
25: **Step 4: Multitask Prediction**26: ${\hat{y}}_{S}\leftarrow \mathrm{Softmax}\left(W_{S}\;\cdot \;\mathrm{GradientReversalLayer}\left(Z_{\mathrm{fusion}}\right)+b_{S}\right)$▷ Site classification27: ${\hat{y}}_{D}\leftarrow \mathrm{Softmax}(W_{D}\;\cdot \;[Z_{\mathrm{ts}}\|Z_{\mathrm{gm}}\|Z_{\mathrm{fusion}}]+b_{D})$▷ MDD classification28:   
29: **Step 5: Multi-Objective Optimization**30: $L_{\mathrm{site}}\leftarrow \mathrm{CrossEntropy}\left({\hat{y}}_{S},y_{S}\right)$31: $L_{\mathrm{mdd}}\leftarrow \mathrm{CrossEntropy}\left({\hat{y}}_{D},y_{D}\right)$32: $L_{\mathrm{total}}\leftarrow \left(1-\beta \right)L_{\mathrm{mdd}}-\beta L_{\mathrm{site}}$

### 3.3. Multitask Learning with Gradient Reversal Layer

**Figure 2:**
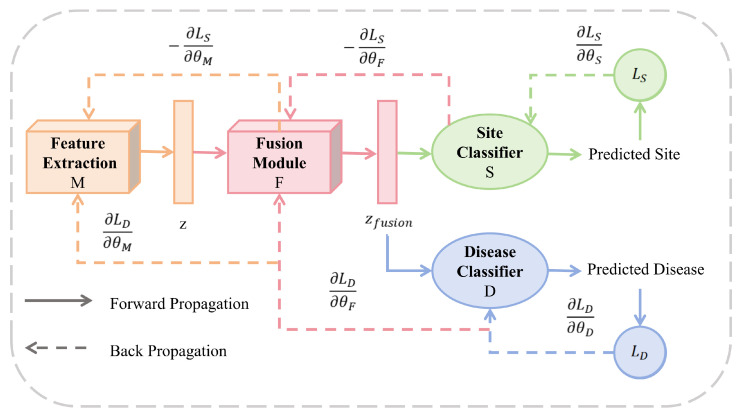
Training workflow and parameter optimization of the MMDD framework. The diagram illustrates the forward propagation (solid arrows) and backward propagation (dotted arrows) paths during model training. The framework consists of four learnable components: feature extraction module M (parameters $\theta_{M}$), fusion module F ($\theta_{F}$), disease classifier D ($\theta_{D}$), and site classifier S ($\theta_{S}$).

We designed a dual-task classification module that achieves cross-site robust diagnosis of MDD through collaborative optimization. The module comprises two classifiers:

Site classifier $C_{1}\left(\;\cdot \;\right)$ employs GRL to eliminate data distribution discrepancies, enabling the model to focus on learning site-invariant features. Inspired by the gradient reversal strategy in [42,48], we employ a Gradient Reversal Layer (GRL) for adversarial training, with the corresponding parameter update rules detailed in Equation (11). This strategy dynamically suppresses site-specific variations in the fused features, effectively eliminating inter-site data distribution discrepancies. Consequently, it guides the model to focus on learning domain-invariant features, ultimately enhancing its generalization capability. The architecture of this site classifier and its associated GRL are illustrated in Figure 2.

(11) $$\begin{array}{cc} \theta_{D} & \leftarrow \theta_{D}-\mu \;\cdot \;\left(1-\beta \right)\frac{\partial L_{D}}{\partial \theta_{D}},\;\theta_{S}\leftarrow \theta_{S}-\mu \;\cdot \;\beta \frac{\partial L_{S}}{\partial \theta_{S}}\\ \theta_{M} & \leftarrow \theta_{M}-\mu \;\cdot \;\left(\left(1-\beta \right)\frac{\partial L_{D}}{\partial \theta_{M}}-\beta \frac{\partial L_{S}}{\partial \theta_{M}}\right),\\ \theta_{F} & \leftarrow \theta_{F}-\mu \;\cdot \;\left(\left(1-\beta \right)\frac{\partial L_{D}}{\partial \theta_{F}}-\beta \frac{\partial L_{S}}{\partial \theta_{F}}\right), \end{array}$$

where $L_{D}$ and $L_{S}$ denote the disease classification loss and the site classification loss, respectively. The hyperparameter β controls the weighting of $L_{S}$ within the total adversarial objective, serving as a trade-off factor that balances the strength of domain invariance against the primary task performance. The model parameters are denoted as follows: $\theta_{F}$ for the feature extraction module, $\theta_{M}$ for the fusion module, $\theta_{D}$ for the disease classifier, and $\theta_{S}$ for the site classifier. The parameters are updated with a learning rate μ.

Disease classifier $C_{2}\left(\;\cdot \;\right)$ performs disease classification by concatenating modality-specific features with cross-modal correlation features. The final output probability $\hat{y}$ of the classifier is computed using Equations (12) and (13):

(12) $${\hat{y}}_{S}=C_{1}\left(\mathrm{GRL}(z_{\mathrm{fusion}})\right)$$

(13) $${\hat{y}}_{D}=C_{2}\left([z_{\mathrm{ts}}\|z_{\mathrm{gm}}\|z_{\mathrm{fusion}}]\right)$$

The model’s performance was evaluated using the mean values from five-fold cross-validation for metrics such as Accuracy, Recall, Area Under the ROC Curve (AUC), F1 score, and Balanced Accuracy (Equation (14)). The classification metrics are calculated as follows:

(14) $$\begin{array}{cc} \mathrm{Accuracy} & =\frac{TP+TN}{TP+TN+FP+FN}\\ \mathrm{Recall} & =\frac{TP}{TP+FN}\\ \mathrm{Precision} & =\frac{TP}{TP+FP}\\ F1\;\mathrm{Score} & =2\times \frac{\mathrm{Precision}\times \mathrm{Recall}}{\mathrm{Precision}+\mathrm{Recall}}\\ \mathrm{FPR} & =\frac{FP}{FP+TN}\\ \mathrm{TPR} & =\frac{TP}{TP+FN}\\ \mathrm{Balanced}\;\mathrm{Accuracy} & =\frac{1}{2}\left(\frac{TP}{TP+FN}+\frac{TN}{TN+FP}\right) \end{array}$$

TP (True Positives): the number of samples that are actually positive and predicted as positive; TN (True Negatives): the number of samples that are actually negative and predicted as negative; FP (False Positives): the number of samples that are actually negative but predicted as positive; FN (False Negatives): the number of samples that are actually positive but predicted as negative. The ROC curve is generated by plotting the points (FPR, TPR). The area beneath this curve is the AUC.

## 4. Results

### 4.1. Datasets

In this study, we utilized the publicly available REST-meta-MDD dataset [49], which aggregates resting-state fMRI and sMRI data from 2428 participants (1300 MDD patients and 1128 HC) across 25 research groups at 17 Chinese hospitals. Specifically, we employed the gray matter volume and time series data provided by the consortium. Ethical approval was obtained from all local institutional review boards, with written informed consent provided by every participant. The dataset exclusively provides pre-processed data; our analysis focused on two modalities: (1) Time series data were extracted from resting-state fMRI using the AAL atlas [50] parcellation (116 regions) and structured as a 116 × T matrix (T = time steps; range: 90–240) due to site-specific acquisition protocols, and (2) gray matter volume (GMV) data derived from structural scans (fixed dimensions: 121 × 145 × 121). Detailed MRI acquisition parameters are documented at https://rfmri.org/REST-meta-MDD, accessed on 17 May 2021.

Since the data manager did not provide demographic information for 48 participants (S4) in the supplied file, our statistical analysis was conducted on the remaining 2380 participants (see details in Table 2). Subsequently, we performed statistical tests to evaluate group differences: a chi-square test was used for sex, while independent samples *t*-tests were applied for age and education level (Table 3).

### 4.2. Data Pre-Processing

The data were pre-processed using an official DPARSF pipeline [51], following the procedure described by Yan et al. [49]. The steps were as follows: first, the initial 10 volumes were discarded to allow for magnetic field stabilization, followed by slice-timing correction and head motion realignment. Next, individual T1-weighted structural images were co-registered to the mean functional image and segmented into gray matter, white matter, and cerebrospinal fluid. Spatial normalization to MNI space was then performed using the DARTEL approach. During nuisance regression, the Friston 24-parameter model was applied to regress out head motion effects. Finally, a temporal bandpass filter (0.01–0.1 Hz) was applied to the time series to extract low-frequency fluctuations.

### 4.3. Implementation Details

In this study, we utilized the REST-meta-MDD dataset to train and evaluate our model. All input data underwent standardized normalization pre-processing. The model was trained using the AdamW optimizer with an initial learning rate (μ) of 0.00005 and a site classification weight (β) of 0.1, a batch size of 16 for 50 epochs, executed on a computing node equipped with four Tesla V100 GPUs interconnected via NVLink. Model performance was assessed through 5-fold cross-validation to ensure statistical reliability, with five key metrics reported as mean values: Accuracy, Balanced Accuracy, Recall, Area Under the Receiver Operating Characteristic Curve (AUC), and F1 Score.

### 4.4. Loss Functions

To optimize the model’s classification performance, we employ the cross-entropy loss [52] function given by Equation (15).

(15) $$L_{ce}=-\sum_{i=1}^{C}y_{i}\log\left(p_{i}\right)$$

where C denotes the number of categories; $y_{i}$ represents whether the sample comes from the $i_{th}$ category. In this work, C = 2 for the primary task (MDD vs. HC), and C = 25 for the auxiliary task (site identification).

The total loss for each sample is illustrated in Equation (16):

(16) $$L_{\mathrm{total}}=\min_{\theta_{D}}\max_{\theta_{S}}\left(1-\beta \right)\;\cdot \;L_{ce}\left(y_{D},p_{D}\right)-\beta \;\cdot \;L_{ce}\left(y_{S},p_{S}\right)$$

where β represents the site loss weight and θ denotes the model parameters. The total loss function incorporates a crucial balancing hyperparameter, β (set to 0.1), which governs the trade-off between the primary MDD classification task and the auxiliary site identification task. All learnable parameters of the model, including the weights of the feature extractors and the fusion module, are represented by θ.

### 4.5. Multimodal Performance Compared with Single-Modal Performance

We conducted a performance comparison between unimodal and multimodal approaches for the HC/MDD classification task (details shown in Table 4). The 3D ResNet model exhibits statistically significant performance advantages in GMV classification, achieving an accuracy of 73.44%. This represents a substantial improvement over other unimodal methods, indicating its strong discriminative capability in capturing depression-related brain structural abnormalities. In contrast, the “LSTM–Transformer” model shows suboptimal classification performance (62.23%), revealing a considerable performance gap compared to GMV-based methods and suggesting inherent limitations in the discriminative power of standalone time series features for MDD classification. Most importantly, our proposed MMDD framework achieves the highest accuracy of 77.76% by synergistically integrating structural and temporal information. This finding conclusively demonstrates the effectiveness of our method in deep representational learning for multimodal neuroimaging data.

### 4.6. Ablation Study

We conducted ablation studies comparing BCAF and GRL-MTL (see Table 5) to investigate their synergistic mechanisms for improving MDD diagnosis. The results showed that the baseline model without any optimization components exhibited clear performance limitations, with high variability (standard deviation > 7%) highlighting its instability. When BCAF was enabled alone, the model demonstrated significant performance improvement with a greatly reduced variance, confirming its effectiveness in establishing dynamic cross-modal feature alignment and enhancing robustness. Although standalone GRL-MTL showed performance degradation, its combination with BCAF achieved optimal results, revealing the complementary roles of these two components: BCAF specializes in cross-modal feature optimization while GRL-MTL focuses on eliminating site-specific biases.

To evaluate the effectiveness of the GRL-MTL strategy in enhancing the cross-site generalization capability of our MDD automated diagnosis model, we designed a leave-one-site-out validation experiment. As detailed in Table 5 and Table 6, we employed a leave-one-site-out cross-validation strategy, where each of the 25 clinical sites was sequentially used as an independent test set, with the remaining 24 sites utilized for model training. Our analysis focuses on performance differences before and after enabling the GRL-MTL strategy. To address class imbalance in the test sets, we applied a sampling strategy in which the majority class was down-sampled to match the size of the minority class. The results demonstrate that, without GRL-MTL, the baseline model exhibited unstable performance across different sites, highlighting significant domain shift issues arising from variations in scanner parameters and heterogeneity in acquisition protocols. In contrast, when GRL-MTL was integrated, the model maintained stable diagnostic performance across all test sites (accuracy: 61.08–65.54%), demonstrating our method’s capability to effectively mitigate inter-site heterogeneity bias in multi-site MDD datasets and enforce learning of site-invariant features.

### 4.7. Visualization

**Figure 3:**
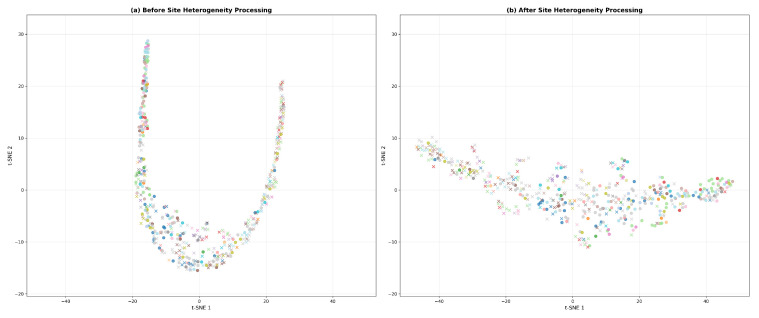
t-SNE visualization of feature distributions (**a**) before and (**b**) after site heterogeneity correction using the GRL-MTL strategy. Markers “o” and “x” represent HC and MDD. Colors denote different acquisition sites.

To investigate the impact of site heterogeneity on feature learning in multi-center data, we first visualized the feature distribution of the baseline fusion model without constraints on site information (Figure 3). The results show that the features are extremely dispersed in the t-SNE [59] space, with data points from different sites widely intermingled, failing to form a discriminative cluster structure. This indicates that the original fused features are severely influenced by site-specific biases rather than reflecting the biological patterns of the disease itself. To address this issue, we introduced the GRL-MTL strategy to learn site-invariant features. As shown in Figure 3, the processed features exhibit a significantly improved clustering trend, where technical variations across sites are effectively suppressed, and the feature distribution demonstrates higher intrinsic consistency. This visual comparison provides an intuitive explanation for the model’s superior classification performance.

## 5. Discussion

**Figure 4:**
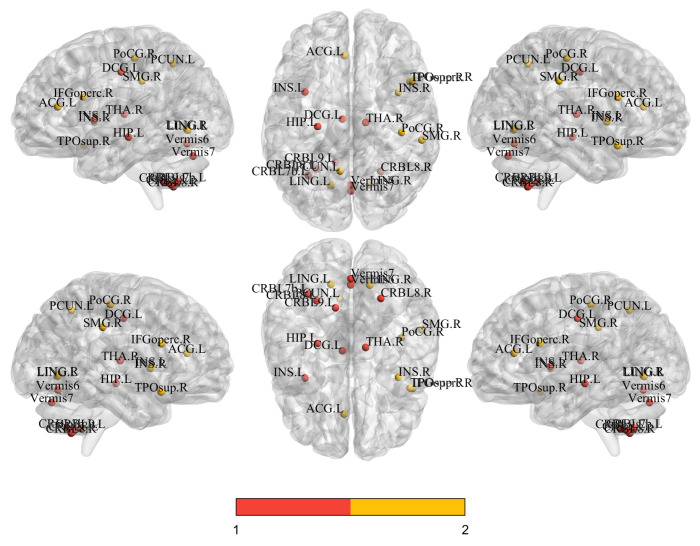
Spatial distribution of the top 14 discriminative features for fMRI (red) and sMRI (orange) identified by interpretability analysis, projected onto standard MNI brain space (coronal, sagittal, and axial views).

To elucidate the key neural mechanisms underpinning the model’s prediction of major depressive disorder (MDD), we employed the SHAP (SHapley Additive exPlanations [60]) methodology to systematically quantify the contribution strengths of temporal sequence dynamic functional features and gray matter volume (GMV) structural features for the classification decision. Through comprehensive multimodal interpretability analysis, we identified brain regions with significant modality-specific influences and further interpreted their potential pathophysiological implications. The feature importance rankings derived from SHAP analysis, along with their statistical significance validated by independent *t*-tests, are presented in Table 7. The corresponding brain network visualization [61] results are shown in Figure 4.

In distinguishing between patients with MDD and HC, the key neuroimaging features based on time series and gray matter volume exhibited distinct yet complementary patterns. The key brain regions identified at the time series level were primarily concentrated in subcortical hubs and the cerebellum, such as “Thalamus_R”, “Hippocampus_L”, and various cerebellar subregions (“Cerebellum_8_R”, “Vermis_7”). This finding is consistent with previous reports of cerebellar abnormalities in MDD patients [62,63]. In contrast, the critical regions identified at the gray matter volume level were clustered in cortical areas associated with higher-order cognition and emotion regulation, including “Cingulum_Mid_L”, “Frontal_Inf_Oper_R”, “Insula_R”, and “Precuneus_L”. The structural alterations in these regions are more directly linked to the core symptoms of depression, such as emotional dysregulation and cognitive dysfunction. Notably, the middle part of the left cingulate gyrus was identified as a significant feature in both modalities. This multimodal comparison suggests that the pathological mechanisms of depression simultaneously involve functional dysregulation in deep brain regions responsible for basic information processing and structural alterations in superficial cortical centers for cognition and emotion.

Collectively, these findings demonstrate a clear bimodal complementarity: while subcortical hubs and the cerebellum primarily manifest as abnormalities in temporal activation patterns, higher-order cognitive and emotion-related cortices predominantly exhibit structural volumetric alterations. These distinct patterns likely reflect divergent neuropathological processes, involving both functional dysregulation in information processing hubs and structural degradation in cognitive–emotional centers. The observed bimodal heterogeneity underscores that reliance on single-modality data may insufficiently capture MDD’s pathological complexity. Future investigations should prioritize the integration of multimodal data to achieve a more comprehensive delineation of the disorder’s neural underpinnings.

Regarding clinical translational potential, these findings hold significant implications. First, the identified multimodal biomarker combinations show promise for objective neuroimaging-based auxiliary diagnostic tools. This would help address the current over-reliance on subjective clinical symptoms for diagnosis, particularly by providing a biological basis for differentiating complex or ambiguous cases. Second, these features can construct disease prediction models, for instance, to identify individuals at high risk, thereby advancing precision medicine. Finally, this deeper understanding of aberrant brain regions (such as the cerebellum) may inspire novel treatment targets, for example, by guiding non-invasive brain stimulation techniques to more precisely target previously overlooked key nodes. Therefore, this study not only deepens our understanding of the neural mechanisms underlying depression but also lays a solid foundation for advancing its clinical diagnosis and treatment towards a more objective and precise paradigm.

This study has several limitations that should be acknowledged. While we conducted statistical tests that revealed significant differences in sex distribution and education level between the MDD and HC groups, these variables were not included as covariates in our primary neuroimaging analyses. It leaves open the possibility that the observed group differences may be partially influenced by these demographic disparities. Future studies should incorporate sex and education as covariates to verify the robustness of our findings.

## 6. Conclusions

This study proposes an innovative multimodal multitask deep learning framework MMDD for the objective diagnosis of major depressive disorder (MDD). By integrating gray matter volume (GMV) features and temporal sequence data, the framework identifies neuropsychiatric biomarkers of depression. In terms of model architecture, a dual-pathway deep neural network is adopted. Specifically, 3D ResNet is employed to extract spatial structural features from GMV, while an LSTM–Transformer hybrid encoder is utilized to model temporal sequence information. Furthermore, an innovative Bidirectional Cross-Attention Fusion (BCAF) mechanism is implemented to achieve dynamic interaction and complementary fusion of spatiotemporal features. To enhance cross-center generalization, a Gradient Reversal Layer-based Multitask Learning (GRL-MTL) strategy is designed to optimize both site-invariant features and disorder-specific signatures.

To uncover the neural mechanisms driving the model’s decisions, we employed SHAP analysis, with key findings validated by two-sample *t*-tests. This approach revealed a clear functional–structural dichotomy: the model’s predictions relied on temporal abnormalities in functional circuits encompassing subcortical hubs and the cerebellum, and on structural volume alterations in higher-order cognitive–emotional cortices. The left middle cingulate gyrus emerged as a convergent hub across both modalities. This bimodal complementarity suggests MDD involves concurrent functional dysregulation in deep brain regions and structural degradation in cortical centers.

In conclusion, this integrative research combines advanced deep learning techniques with interpretability analysis. Beyond establishing a novel paradigm for developing robust and generalizable objective diagnostic tools for depression, the study significantly advances our understanding of the multimodal neural mechanisms underlying MDD, with profound clinical implications for advancing precision medicine in psychiatric disorders.

## Figures and Tables

**Table 2:** Demographic Characteristics of MDD and HC Groups Across Study Sites.

Site	MDD					HC				
	Age	Education	Female	Male	Count	Age	Education	Female	Male	Count
S1	31.72 ± 8.19	13.8 ± 2.94	43	31	74	31.80 ± 8.99	15.23 ± 2.26	42	32	74
S2	43.87 ± 12.94	10.30 ± 4.30	24	6	30	44.6 ± 12.39	10.40 ± 4.75	21	9	30
S3	20.37 ± 1.84	0	16	11	27	20.41 ± 1.62	0	23	14	37
S5	33.08 ± 5.91	0	5	8	13	33.00 ± 4.96	0	6	5	11
S6	30.8 ± 10.94	12.73 ± 4.06	9	6	15	28.47 ± 10.89	14.67 ± 3.54	9	6	15
S7	42.58 ± 11.85	10.37 ± 4.63	23	15	38	41.43 ± 13.41	13.16 ± 6.07	30	19	49
S8	28.59 ± 10.85	10.35 ± 3.73	48	27	75	28.68 ± 11.10	12.63 ± 2.85	45	30	75
S9	28.10 ± 8.83	13.28 ± 2.85	27	23	50	28.92 ± 8.59	15.72 ± 3.04	19	31	50
S10	33.00 ± 10.63	11.28 ± 3.39	28	22	50	30.15 ± 11.61	11.64 ± 4.24	13	20	33
S11	30.44 ± 10.87	11.25 ± 2.7	19	13	32	32.83 ± 9.98	15.17 ± 3.49	19	10	29
S12	34.53 ± 9.69	12.78 ± 2.76	26	6	32	31.33 ± 2.25	13.67 ± 0.82	1	5	6
S13	32.28 ± 9.30	13.64 ± 2.08	14	11	25	34.00 ± 10.50	13.12 ± 2.32	11	6	17
S14	30.53 ± 7.09	13.72 ± 3.35	43	21	64	29.59 ± 5.00	14.59 ± 2.82	17	15	32
S15	46.92 ± 15.42	9.30 ± 4.91	34	16	50	46.48 ± 17.65	11.80 ± 4.45	24	26	50
S16	31.06 ± 9.00	14.55 ± 5.01	15	16	31	31.68 ± 10.33	0	18	13	31
S17	20.98 ± 3.38	13.13 ± 1.74	31	16	47	20.32 ± 2.11	13.68 ± 1.57	30	14	44
S18	31.43 ± 6.98	12.29 ± 3.99	15	6	21	30.10 ± 7.52	12.40 ± 3.14	12	8	20
S19	38.25 ± 11.51	8.78 ± 3.90	37	14	51	35.86 ± 10.27	9.94 ± 3.84	18	18	36
S20	38.74 ± 13.65	10.78 ± 3.61	183	99	282	39.64 ± 15.87	12.97 ± 3.94	164	87	251
S21	34.71 ± 12.63	11.65 ± 2.89	48	38	86	36.13 ± 12.64	12.83 ± 2.59	39	31	70
S22	33.57 ± 10.26	11.53 ± 2.78	16	14	30	24.35 ± 7.07	13.30 ± 2.13	8	12	20
S23	25.03 ± 8.63	12.81 ± 3.40	17	15	32	32.17 ± 13.10	13.97 ± 4.87	19	11	30
S24	31.59 ± 8.48	12.69 ± 4.00	24	8	32	31.61 ± 7.49	14.42 ± 1.88	20	11	31
S25	65.60 ± 6.75	11.28 ± 2.91	68	21	89	69.63 ± 5.86	12.83 ± 3.01	34	29	63

**Table 3:** Comparison of Demographic Characteristics Between MDD and HC Groups.

Characteristic	Total (*n* = 2380)	MDD (*n* = 1276)	Control (*n* = 1104)	Statistics	*p*-Value
**Age**	36.19 ± 15.11	36.23 ± 14.62	36.15 ± 15.67	t = 0.1274	0.8986
Range	0.00–82.00	12.00–82.00	0.00–80.00	–	–

**Education**	11.71 ± 4.61	11.25 ± 4.21	12.25 ± 4.98	t = −5.2492	<0.001 ***
Range	0.00–23.00	0.00–21.00	0.00–23.00	–	–

**Gender**				$\chi^{2}$ = 7.4757	0.0063 *
Male (*n*/%)	925 (38.9%)	463 (36.3%)	462 (41.8%)	–	–
Female (*n*/%)	1455 (61.1%)	813 (63.7%)	642 (58.2%)	–	–

Note: Data are presented as mean ± standard deviation; * *p* < 0.05, *** *p* < 0.001.

**Table 4:** Model performance comparison across gray matter volume (GMV) and time series (TS) modalities (%, mean; ± std).

Modality	Model	Accuracy	Recall	AUC	F1 Score
GMV	SVM [53]	63.97 ± 7.15	61.92 ± 13.07	70.40 ± 10.21	64.23 ± 9.42
	MLP [54]	54.98 ± 1.96	83.06 ± 8.51	51.90 ± 2.54	66.28 ± 1.64
	3D CNN [55]	54.61 ± 1.65	89.06 ± 8.41	53.08 ± 0.72	67.62 ± 2.89
	3D DenseNet [56]	55.97 ± 0.21	82.33 ± 3.21	54.26 ± 0.75	66.72 ± 0.75
	Auto Encoder [57]	73.12±5.23	73.12±5.23	**84.59 ± 3.53**	72.81±5.56
	Swin Transformer [58]	55.56±2.40	**92.62 ± 8.85**	54.46±1.71	68.94±2.55
	3D ResNet [47]	**73.44 ± 2.00**	75.17 ± 4.04	81.02 ± 2.67	**75.17 ± 1.53**
TS	SVM	57.37 ± 1.08	61.15 ± 2.54	58.53 ± 1.95	60.55 ± 1.48
	MLP	51.89 ± 1.88	51.89 ± 1.88	54.85 ± 2.33	51.85 ± 2.00
	CNN	62.07 ± 3.11	62.07 ± 3.11	**67.48 ± 1.27**	61.87 ± 3.31
	CNN–Transformer	60.92 ± 2.62	60.92 ± 2.62	64.31 ± 4.29	59.86 ± 3.52
	CNN-LSTM	60.58 ± 2.26	60.58 ± 2.26	66.46 ± 3.22	60.18 ± 2.17
	Auto Encoder	54.98±1.80	54.98±1.80	56.45±1.71	54.98±1.78
	Swin Transformer	53.62±2.83	53.62±2.83	49.65±2.37	40.57±7.19
	LSTM–Transformer	**62.23 ± 1.84**	**62.23 ± 1.84**	66.30 ± 1.84	**62.13 ± 1.88**
GMV+TS	SVM	72.65 ± 1.26	73.17 ± 2.15	**80.37 ± 1.49**	74.09 ± 1.64
	CNN	69.44 ± 1.47	73.46 ± 7.36	69.13 ± 1.92	71.90 ± 1.89
	3D ResNet+	75.08 ± 1.58	77.00 ± 3.65	74.94 ± 1.80	76.79 ± 1.17
	CNN–Transformer				
	Auto Encoder	58.32±1.11	58.29±1.42	57.79±0.97	58.32±1.11
	Swin Transformer	53.54±0.05	53.54±0.05	52.29±1.74	37.34±0.06
	Proposed Work	**77.76 ± 1.02**	**83.23 ± 7.06**	77.34 ± 0.92	**79.92 ± 1.91**

Bold values indicate the best performance in each metric column.

**Table 5:** Cross-Site Generalization of GRL-MTL for MDD Diagnosis on REST-meta-MDD.

Site	MDD/HC	GRL-MTL	Performance Metrics (%, Mean ± Std)				
			Accuracy	Bal Accuracy	Recall	AUC	F1 Score
S1	74/74		62.62 ± 1.49	62.62 ± 1.49	69.53 ± 7.05	62.62 ± 1.49	64.93 ± 3.06
		✓	65.54 ± 1.35	65.54 ± 1.35	63.06 ± 9.96	65.54 ± 1.35	64.45 ± 2.92
S2	30/30		57.00 ± 5.16	57.00 ± 5.16	60.67 ± 10.03	57.00 ± 5.16	58.34 ± 2.03
		✓	59.30 ± 2.20	59.30 ± 2.20	63.30 ± 6.70	59.30 ± 2.20	60.80 ± 2.50
S3	27/27		47.78 ± 2.35	47.78 ± 2.35	80.00 ± 8.25	47.78 ± 2.35	60.38 ± 2.56
		✓	50.00 ± 1.85	50.00 ± 1.85	67.41 ± 25.71	50.00 ± 1.85	55.31 ± 12.72
S4	24/24		89.58 ± 4.42	89.58 ± 4.42	87.50 ± 6.59	89.58 ± 4.42	89.34 ± 4.53
		✓	92.50 ± 2.80	92.50 ± 2.80	92.50 ± 3.49	92.50 ± 2.80	92.53 ± 2.68
S5	11/11		57.28 ± 4.07	57.28 ± 4.07	45.45 ± 11.13	57.28 ± 4.07	50.96 ± 7.60
		✓	59.09 ± 5.56	59.09 ± 5.56	65.46 ± 7.61	59.09 ± 5.56	61.47 ± 5.26
S6	15/15		50.67 ± 1.47	50.67 ± 1.47	61.33 ± 36.93	50.67 ± 1.47	51.22 ± 15.56
		✓	51.33 ± 1.83	51.33 ± 1.83	87.47 ± 14.41	51.33 ± 1.83	54.58 ± 24.06
S7	38/38		58.16 ± 5.77	58.16 ± 5.77	74.21 ± 11.97	58.16 ± 5.77	63.73 ± 5.30
		✓	63.95 ± 2.88	63.95 ± 2.88	62.63 ± 9.74	63.95 ± 2.88	63.27 ± 3.11
S8	75/75		57.17 ± 1.14	57.17 ± 1.14	48.33 ± 6.92	57.17 ± 1.14	52.82 ± 4.08
		✓	62.89 ± 1.68	62.89 ± 1.68	64.89 ± 4.68	62.89 ± 1.68	63.57 ± 2.39
S9	50/50		58.40 ± 7.16	58.40 ± 7.16	59.60 ± 4.33	58.40 ± 7.16	53.60 ± 3.71
		✓	62.20 ± 1.30	62.20 ± 1.30	57.20 ± 0.52	62.20 ± 1.30	58.97 ± 7.02
S10	33/33		58.18 ± 2.30	58.18 ± 2.30	55.76 ± 15.24	58.18 ± 2.30	56.47 ± 4.83
		✓	59.09 ± 3.86	59.09 ± 3.86	58.19 ± 4.98	59.09 ± 3.86	58.69 ± 3.90
S11	29/29		54.48 ± 4.15	54.48 ± 4.15	67.59 ± 11.07	54.48 ± 4.15	59.46 ± 6.07
		✓	55.86 ± 3.97	55.86 ± 3.97	68.27 ± 15.69	55.86 ± 3.97	60.14 ± 7.12
S12	6/6		56.67 ± 6.97	56.67 ± 6.97	66.67 ± 20.41	56.67 ± 6.97	59.89 ± 7.43
		✓	61.67 ± 4.57	61.67 ± 4.57	80.00 ± 7.45	61.67 ± 4.57	67.55 ± 4.12
S13	17/17		53.53 ± 3.83	53.53 ± 3.83	84.71 ± 11.47	53.53 ± 3.83	64.46 ± 1.84
		✓	57.64 ± 2.63	57.64 ± 2.63	62.35 ± 5.26	57.64 ± 2.63	59.48 ± 3.18
S14	32/32		61.61 ± 3.10	61.61 ± 3.10	61.29 ± 5.10	61.61 ± 3.10	61.45 ± 2.82
		✓	84.69 ± 2.32	84.69 ± 2.32	85.00 ± 10.69	84.69 ± 2.32	84.54 ± 3.34
S15	50/50		57.40 ± 3.36	57.40 ± 3.36	70.80 ± 8.32	57.40 ± 3.36	62.31 ± 3.44
		✓	60.20 ± 2.39	60.20 ± 2.39	71.20 ± 5.02	60.20 ± 2.39	64.12 ± 1.99
S16	31/31		56.13 ± 2.10	56.13 ± 2.10	67.10 ± 8.35	56.13 ± 2.10	60.29 ± 3.76
		✓	67.42 ± 3.10	67.42 ± 3.10	76.78 ± 3.53	67.42 ± 3.10	70.24 ± 1.68
S17	44/44		51.36 ± 1.48	51.36 ± 1.48	78.18 ± 12.61	51.36 ± 1.48	61.40 ± 2.99
		✓	52.73 ± 1.30	52.73 ± 1.30	69.09 ± 9.98	52.73 ± 1.30	59.14 ± 3.56
S18	20/20		51.50 ± 2.24	51.50 ± 2.24	74.00 ± 7.42	51.50 ± 2.24	60.29 ± 3.35
		✓	53.00 ± 3.26	53.00 ± 3.26	68.00 ± 18.91	53.00 ± 3.26	58.42 ± 5.55
S19	36/36		58.89 ± 4.00	58.89 ± 4.00	58.34 ± 10.94	58.89 ± 4.00	58.36 ± 5.40
		✓	60.55 ± 1.58	60.55 ± 1.58	62.78 ± 5.04	60.55 ± 1.58	61.34 ± 2.80
S20	251/251		52.39 ± 2.19	52.39 ± 2.19	65.50 ± 12.20	52.39 ± 2.19	57.55 ± 4.41
		✓	54.94 ± 0.74	54.94 ± 0.74	71.16 ± 10.90	54.94 ± 0.74	60.96 ± 3.76
S21	70/70		61.08 ± 1.78	61.08 ± 1.78	53.57 ± 6.43	61.08 ± 1.78	57.75 ± 1.82
		✓	62.32 ± 0.59	62.32 ± 0.59	71.07 ± 8.59	62.32 ± 0.59	65.14 ± 2.61
S22	20/20		54.50 ± 4.81	54.50 ± 4.81	43.00 ± 20.80	54.50 ± 4.81	45.66 ± 15.61
		✓	65.00 ± 3.95	65.00 ± 3.95	64.00 ± 4.18	65.00 ± 3.95	64.69 ± 2.71
S23	30/30		57.33 ± 2.79	57.33 ± 2.79	72.00 ± 4.47	57.33 ± 2.79	62.78 ± 1.81
		✓	62.00 ± 2.17	62.00 ± 2.17	75.33 ± 5.06	62.00 ± 2.17	66.42 ± 2.74
S24	31/31		55.81 ± 1.84	55.81 ± 1.84	59.36 ± 8.10	55.81 ± 1.84	57.14 ± 3.08
		✓	61.61 ± 3.10	61.61 ± 3.10	61.29 ± 5.10	61.61 ± 3.10	61.45 ± 2.82
S25	63/63		62.70 ± 1.59	62.70 ± 1.59	69.31 ± 7.50	62.70 ± 1.59	64.93 ± 1.85
		✓	64.55 ± 2.46	64.55 ± 2.46	53.97 ± 1.83	64.55 ± 2.46	58.41 ± 1.76

The checkmark (✓) indicates that the GRL-MTL strategy was applied; its absence indicates the strategy was not used.

**Table 6:** Ablation Study of BCAF (B) and GRL-MTL (G) Components for MDD Diagnosis on REST-meta-MDD (5-fold CV).

Method	Performance Metrics (%, Mean ± Std)			
	Accuracy	Recall	AUC	F1 Score
-	67.46 ± 7.11	70.77 ± 9.02	67.21 ± 6.97	69.79 ± 7.18
B	75.41 ± 1.45	82.46 ± 5.45	74.88 ± 1.29	78.14 ± 2.08
G	65.16 ± 2.19	65.85 ± 3.40	65.11 ± 2.27	66.91 ± 2.17
B+G	**77.76 ± 1.02**	**83.23 ± 7.06**	**77.34 ± 0.92**	**79.92 ± 1.91**

**Table 7:** Top 10 Discriminative Brain Regions Identified by SHAP Analysis and Two-Sample *t*-tests.

Time Series			Gray Matter Volume		
Region	SHAP-Value	* p * -Value	Region	SHAP-Value	* p * -Value
Thalamus_R	−0.0038	0.0085	Cingulum_Mid_L	−0.0001	0.0003
Hippocampus_L	−0.0070	0.0118	Frontal_Inf_Oper_R	0.0018	0.0002
Cerebellum_8_L	−0.0080	0.0149	Precuneus_L	0.0007	0.0001
Cerebellum_7b_L	0.0023	0.0195	Insula_R	−0.0014	0.0001
Cerebellum_8_R	0.0041	0.0236	Lingual_R	−0.0006	0.0002
Vermis_7	0.0224	0.0249	Cingulum_Ant_L	−0.0030	0.0011
Vermis_6	0.0354	0.0322	Lingual_L	0.0006	0.0012
Cerebellum_9_L	−0.0055	0.0328	Postcentral_R	0.0002	0.0033
Insula_L	0.0180	0.0432	SupraMarginal_R	0.0002	0.0043
Cingulum_Mid_L	0.0049	0.0511	Temporal_Pole_Sup_R	−0.0003	0.0075

## Data Availability

The data analyzed in this study were obtained from a publicly available dataset. The dataset can be accessed here: https://rfmri.org/REST-meta-MDD (accessed on 17 May 2021).

## References

[1] Marx W., Penninx B., Solmi M., Furukawa T., Firth J., Carvalho A., Berk M. (2023). Major depressive disorder. Nat. Rev. Dis. Prim..

[2] (2025). World Health Organization Depressive Disorder (Depression). https://www.who.int/news-room/fact-sheets/detail/depression.

[3] Als T., Kurki M., Grove J., Voloudakis G., Therrien K., Tasanko E., Nielsen T., Naamanka J., Veerapen K., Levey D. (2023). Depression pathophysiology, risk prediction of recurrence and comorbid psychiatric disorders using genome-wide analyses. Nat. Med..

[4] Li Z., Li W., Wei Y., Gui G., Zhang R., Liu H., Chen Y., Jiang Y. (2021). Deep learning based automatic diagnosis of first-episode psychosis, bipolar disorder and healthy controls. Comput. Med. Imaging Graph..

[5] Zhang Q., Long Y., Cai H., Chen Y. (2024). Lightweight neural network for Alzheimer’s disease classification using multi-slice sMRI. Magn. Reson. Imaging.

[6] Abrol A., Fu Z., Du Y., Calhoun V. Multimodal data fusion of deep learning and dynamic functional connectivity features to predict Alzheimer’s disease progression. Proceedings of the 2019 41st Annual International Conference of The IEEE Engineering in Medicine and Biology Society (EMBC).

[7] Kan X., Cui H., Lukemire J., Guo Y., Yang C. (2022). Fbnetgen: Task-aware gnn-based fmri analysis via functional brain network generation. Int. Conf. Med. Imaging Deep. Learn..

[8] Hu X., Cheng B., Tang Y., Long T., Huang Y., Li P., Song Y., Song X., Li K., Yin Y. (2024). Gray matter volume and corresponding covariance connectivity are biomarkers for major depressive disorder. Brain Res..

[9] Wu Y., Kong L., Yang A., Xin K., Lu Y., Yan X., Liu W., Zhu Y., Guo Y., Jiang X. (2023). Gray matter volume reduction in orbitofrontal cortex correlated with plasma glial cell line-derived neurotrophic factor (GDNF) levels within major depressive disorder. Neuroimage Clin..

[10] Luo Y., Chen W., Zhan L., Qiu J., Jia T. (2024). Multi-feature concatenation and multi-classifier stacking: An interpretable and generalizable machine learning method for MDD discrimination with rsfMRI. NeuroImage.

[11] Liu S., Gui R. (2024). Fusing multi-scale fMRI features using a brain-inspired multi-channel graph neural network for major depressive disorder diagnosis. Biomed. Signal Process. Control.

[12] Rafie Z., Talab M.S., Koor B.E.Z., Garavand A., Salehnasab C., Ghaderzadeh M. (2025). Leveraging XGBoost and explainable AI for accurate prediction of type 2 diabetes. BMC Public Health.

[13] Tajvidi Asr R., Rahimi M., Hossein Pourasad M., Zayer S., Momenzadeh M., Ghaderzadeh M. (2024). Hematology and hematopathology insights powered by machine learning: Shaping the future of blood disorder management. Iran. J. Blood Cancer.

[14] Fortin J., Parker D., Tunç B., Watanabe T., Elliott M., Ruparel K., Roalf D., Satterthwaite T., Gur R., Gur R. (2017). Harmonization of multi-site diffusion tensor imaging data. Neuroimage.

[15] Guan H., Liu Y., Yang E., Yap P., Shen D., Liu M. (2021). Multi-site MRI harmonization via attention-guided deep domain adaptation for brain disorder identification. Med. Image Anal..

[16] Mengi M., Malhotra D. (2024). USMDA: Unsupervised Multisource Domain Adaptive ADHD prediction model using neuroimaging. Knowl. Based Syst..

[17] Dai M., Su J., Fan Z., Wang C., Peng L., Hu D., Zeng L. (2025). Feature and Semantic Matching Multi-Source Domain Adaptation for Diagnostic Classification of Neuropsychiatric Disorders. IEEE Trans. Cogn. Dev. Syst..

[18] Noman F., Ting C., Kang H., Phan R., Ombao H. (2024). Graph autoencoders for embedding learning in brain networks and major depressive disorder identification. IEEE J. Biomed. Health Inform..

[19] Wang Z., Zhou X., Gui Y., Liu M., Lu H. (2023). Multiple measurement analysis of resting-state fMRI for ADHD classification in adolescent brain from the ABCD study. Transl. Psychiatry.

[20] Muksimova S., Umirzakova S., Iskhakova N., Khaitov A., Im Cho Y. (2025). Advanced convolutional neural network with attention mechanism for Alzheimer’s disease classification using MRI. Comput. Biol. Med..

[21] Bai S., Kolter J., Koltun V. (2018). An empirical evaluation of generic convolutional and recurrent networks for sequence modeling. arXiv.

[22] Xin J., Wang A., Guo R., Liu W., Tang X. (2023). CNN and swin-transformer based efficient model for Alzheimer’s disease diagnosis with sMRI. Biomed. Signal Process. Control.

[23] Kim P., Kwon J., Joo S., Bae S., Lee D., Jung Y., Yoo S., Cha J., Moon T. (2023). Swift: Swin 4d fmri transformer. Adv. Neural Inf. Process. Syst..

[24] Qu J., Qu Y., Bai X., Xu L., Wang H., Liu G. Multimodal Medical Image Feature Representation and Fusion for AD Early Diagnosis. Proceedings of the 2024 IEEE International Conference On Bioinformatics And Biomedicine (BIBM).

[25] Zhu M., Quan Y., He X. (2023). The classification of brain network for major depressive disorder patients based on deep graph convolutional neural network. Front. Hum. Neurosci..

[26] Gallo S., El-Gazzar A., Zhutovsky P., Thomas R.M., Javaheripour N., Li M., Bartova L., Bathula D., Dannlowski U., Davey C. (2023). Functional connectivity signatures of major depressive disorder: Machine learning analysis of two multicenter neuroimaging studies. Mol. Psychiatry.

[27] Liu C., Shan S., Ding X., Wang H., Jiao Z. (2025). FGDN: A Federated Graph Convolutional Network framework for multi-site major depression disorder diagnosis. Comput. Med. Imaging Graph..

[28] Su S., Ning Y., Guo Z., Yang W., Zhu M., Zhou Q., He X. (2025). Classification of Major Depressive Disorder Using Graph Attention Mechanism with Multi-Site rs-fMRI Data. Neuroinformatics.

[29] Zhao T., Zhang G. (2024). Enhancing major depressive disorder diagnosis with dynamic-static fusion graph neural networks. IEEE J. Biomed. Health Inform..

[30] Tan Y.-F., Loo J.-Y., Ting C.-M., Noman F., Phan R.C.-W., Ombao H. Brainfc-cgan: A conditional generative adversarial network for brain functional connectivity augmentation and aging synthesis. Proceedings of the ICASSP 2024–2024 IEEE International Conference on Acoustics, Speech and Signal Processing (ICASSP).

[31] Zhang Y., Liu X., Zhang Z. DDN-net: Deep residual shrinkage denoising networks with channel-wise adaptively soft thresholds for automated major depressive disorder identification. Proceedings of the ICASSP 2024–2024 IEEE International Conference on Acoustics, Speech and Signal Processing (ICASSP).

[32] Fang C., Liang F., Li T., Guan F. (2024). Learning modality consistency and difference information with multitask learning for multimodal sentiment analysis. Future Internet.

[33] Wang J., Li T., Sun Q., Guo Y., Yu J., Yao Z., Hou N., Hu B. (2023). Automatic diagnosis of major depressive disorder using a high-and low-frequency feature fusion framework. Brain Sci..

[34] Gupta C., Khullar V., Goyal N., Saini K., Baniwal R., Kumar S., Rastogi R. (2023). Cross-silo, privacy-preserving, and lightweight federated multimodal system for the identification of major depressive disorder using audio and electroencephalogram. Diagnostics.

[35] Wu P., Wang Z., Zheng B., Li H., Alsaadi F., Zeng N. (2023). AGGN: Attention-based glioma grading network with multi-scale feature extraction and multi-modal information fusion. Comput. Biol. Med..

[36] Srivastava S., Sharma G. Omnivec2-a novel transformer based network for large scale multimodal and multitask learning. Proceedings of the IEEE/CVF Conference on Computer Vision and Pattern Recognition.

[37] Liu M., Wang Z., Liu F. MDDMamba: A Model for Multi-Modal Depression Detection with a Memory-Saving Cross-Modal Attention Mechanism Based on Mamba. Proceedings of the 2024 IEEE International Conference On Bioinformatics and Biomedicine (BIBM).

[38] Bi X., Luo S., Jiang S., Wang Y., Xing Z., Xu L. (2023). Explainable and programmable hypergraph convolutional network for imaging genetics data fusion. Inf. Fusion.

[39] Xu C., Li J., Wang Y., Wang L., Wang Y., Zhang X., Liu W., Chen J., Vatian A., Gusarova N. (2024). SiMix: A domain generalization method for cross-site brain MRI harmonization via site mixing. NeuroImage.

[40] Svanera M., Savardi M., Signoroni A., Benini S., Muckli L. (2024). Fighting the scanner effect in brain MRI segmentation with a progressive level-of-detail network trained on multi-site data. Med. Image Anal..

[41] Ren Y., Ma Z., Ding Z., Yang R., Li X., He X., Liu T. (2025). SFPGCL: Specificity-preserving federated population graph contrastive learning for multi-site ASD identification using rs-fMRI data. Comput. Med. Imaging Graph..

[42] Yu W., Huang Z., Zhang J., Shan H. (2023). SAN-Net: Learning generalization to unseen sites for stroke lesion segmentation with self-adaptive normalization. Comput. Biol. Med..

[43] Zheng G., Zheng W., Zhang Y., Wang J., Chen M., Wang Y., Cai T., Yao Z., Hu B. (2023). An attention-based multi-modal mri fusion model for major depressive disorder diagnosis. J. Neural Eng..

[44] Xi W., Guo Z., Mei T., Zhou Q., Xue M., Yang W., Guo Y., He X. (2025). Random walk-based node feature learning for major depressive disorder identification through multi-site rs-fMRI data. Hum. Brain Mapp..

[45] Graves A., Schmidhuber J. (2005). Framewise phoneme classification with bidirectional LSTM and other neural network architectures. Neural Netw..

[46] Vaswani A., Shazeer N., Parmar N., Uszkoreit J., Jones L., Gomez A., Kaiser Ł., Polosukhin I. (2017). Attention is all you need. Adv. Neural Inf. Process. Syst..

[47] Hara K., Kataoka H., Satoh Y. Can spatiotemporal 3d cnns retrace the history of 2d cnns and imagenet?. Proceedings of the IEEE Conference On Computer Vision And Pattern Recognition.

[48] Ganin Y., Lempitsky V. (2015). Unsupervised domain adaptation by backpropagation. Int. Conf. Mach. Learn..

[49] Yan C.G., Chen X., Li L., Castellanos F.X., Bai T.J., Bo Q.J., Cao J., Chen G.M., Chen N.X., Chen W. (2019). Reduced default mode network functional connectivity in patients with recurrent major depressive disorder. Proc. Natl. Acad. Sci. USA.

[50] Tzourio-Mazoyer N., Landeau B., Papathanassiou D., Crivello F., Etard O., Delcroix N., Mazoyer B., Joliot M. (2002). Automated anatomical labeling of activations in SPM using a macroscopic anatomical parcellation of the MNI MRI single-subject brain. Neuroimage.

[51] Yan C., Wang X., Zuo X., Zang Y. (2016). DPABI: Data processing & analysis for (resting-state) brain imaging. Neuroinformatics.

[52] Murphy K. (2012). Machine Learning: A Probabilistic Perspective.

[53] Cortes C., Vapnik V. (1995). Support-vector networks. Mach. Learn..

[54] Rumelhart D., Hinton G., Williams R. (1986). Learning representations by back-propagating errors. Nature.

[55] Ji S., Xu W., Yang M., Yu K. (2012). 3D convolutional neural networks for human action recognition. IEEE Trans. Pattern Anal. Mach. Intell..

[56] Huang G., Liu Z., Van Der Maaten L., Weinberger K. Densely connected convolutional networks. Proceedings of the IEEE Conference on Computer Vision and Pattern Recognition.

[57] Hinton G.E., Salakhutdinov R.R. (2006). Reducing the dimensionality of data with neural networks. Science.

[58] Liu Z., Lin Y., Cao Y., Hu H., Wei Y., Zhang Z., Lin S., Guo B. Swin transformer: Hierarchical vision transformer using shifted windows. Proceedings of the IEEE/CVF International Conference on Computer Vision.

[59] Maaten L., Hinton G. (2008). Visualizing Data using t-SNE. J. Mach. Learn. Res..

[60] Lundberg S., Lee S. A unified approach to interpreting model predictions. Proceedings of the 31st International Conference On Neural Information Processing Systems.

[61] Xia M., Wang J., He Y. (2013). BrainNet Viewer: A network visualization tool for human brain connectomics. PloS ONE..

[62] Schmahmann J., Sherman J. (1998). The cerebellar cognitive affective syndrome. Brain A J. Neurol..

[63] Alalade E., Denny K., Potter G., Steffens D., Wang L. (2011). Altered cerebellar-cerebral functional connectivity in geriatric depression. PLoS ONE.

